# Retinal Nerve Fiber Layer Thinning Is Associated With Brain Atrophy: A Longitudinal Study in Nondemented Older Adults

**DOI:** 10.3389/fnagi.2019.00069

**Published:** 2019-04-11

**Authors:** Zhongyong Shi, Hailin Zheng, Jingxiao Hu, Lijuan Jiang, Xinyi Cao, Yupeng Chen, Xinchun Mei, Chunbo Li, Yuan Shen

**Affiliations:** ^1^Department of Psychiatry, Shanghai 10th People’s Hospital, Tongji University, Shanghai, China; ^2^Anesthesia and Brain Research Institute, Tongji University School of Medicine, Shanghai, China; ^3^Shanghai Key Laboratory of Psychiatric Disorders, Shanghai Mental Health Center, Shanghai Jiao Tong University School of Medicine, Shanghai, China; ^4^Institute of Psychology and Behavioral Science, Shanghai Jiao Tong University, Shanghai, China

**Keywords:** retinal nerve fiber layer, optical coherence tomography, brain atrophy, magnetic resonance imaging, Alzheimer’s disease

## Abstract

**Backgrounds**: Abnormal retinal nerve fiber layer (RNFL) thickness has been observed in patients with Alzheimer’s disease (AD) and therefore suggested to be a potential biomarker of AD. However, whether the changes in RNFL thickness are associated with the atrophy of brain structure volumes remains unknown. We, therefore, set out a prospective investigation to determine the association between longitudinal changes of RNFL thickness and brain atrophy in nondemented older participants over a period of 12 months.

**Materials and Methods**: We measured the RNFL thickness using optical coherence tomography (OCT) and brain structure volumes by 3T magnetic resonance imaging (MRI) before and after 12 months. Cognitive function was assessed using the Chinese version of Mini-Mental State Examination (CMMSE) and Repeatable Battery for the Assessment of Neurological Status. Associations among the changes of RNFL, brain structures and cognitive function were analyzed with *Spearman* correlation and multiple linear regression models adjusting for the confounding factors.

**Results**: Fifty old participants were screened and 40 participants (mean age 71.8 ± 3.9 years, 60% were male) were enrolled at baseline. Among them, 28 participants completed the follow-up assessments. The average reduction of RNFL thickness was inversely associated with the decrease of central cingulate cortex volume after the adjustment of age and total intracranial volume (*β* = −0.41, *P* = 0.039). Specifically, the reduction of RNFL thickness in the inferior, not other quadrants, was independently associated with the decline of central cingulate cortex volume after the adjustment (*β* = −0.52, *P* = 0.006). Moreover, RNFL thinning, central cingulate cortex atrophy and the aggregation of white matter hyperintensities (WMH) were found associated with episodic memory in these older adults with normal cognition.

**Conclusions**: RNFL thinning was associated with cingulate cortex atrophy and episodic memory decline in old participants. The longitudinal changes of RNFL thickness are suggested to be a useful complementary index of neurocognitive aging or neurodegeneration.

## Introduction

Alzheimer’s disease (AD) begins with a long asymptomatic period when only its neuropathogenesis progresses (Price and Morris, 1999; Yaffe et al., 2012; Jack et al., 2013). It has been emphasized that the early detection of AD through biomarkers will help target early intervention, leading to the potential delay of AD onset (Sperling et al., 2014; Soldan et al., 2016). The hallmark characteristics of AD at the present time include aberrant reduced β-amyloid protein (Aβ) in cerebrospinal fluid (CSF; Visser et al., 2009), increased amyloid and Tau tracer retention revealed by positron emission tomography (PET; Tosun et al., 2017), and hippocampus atrophy as detected by magnetic resonance imaging (MRI; Hampel et al., 2008). However, the clinical applicability of these biomarkers is limited due to either invasive or expensive. Therefore, there is an urgent need to establish a noninvasive and less expensive AD biomarker to identify high-risk individuals and initiate interventions at an early stage (Bateman et al., 2012).

The retina and optic nerve extend from the diencephalon during embryonic development and are thus considered as part of the central nervous system, which can be directly visualized (London et al., 2013). The retinal nerve fiber layer (RNFL) is composed primarily of ganglion cell axons before they coalesce in the optic nerve. Early postmortem studies by Hinton et al. (1986) demonstrated the axonal degeneration of the optic nerve and decreased thickness of the RNFL in AD patients. Since then, noninvasive optical imaging techniques have increasingly been used to study the ocular disorders and the ocular involvement in neurological diseases (Kirbas et al., 2013b; Saidha et al., 2013; Bussel et al., 2014; Doustar et al., 2017). In particular, optical coherence tomography (OCT), a rapid and high-resolution imaging tool, enables precise and accurate measurement of *in vivo* RNFL thickness (Huang et al., 1991).

Evidences have suggested that patients with AD or mild cognitive impairment (MCI) demonstrated thinner RNFL as compared with heathy age-matched controls (He et al., 2012; Thomson et al., 2015; den Haan et al., 2017), indicating that RNFL thinning is an early event in AD pathology. RNFL thickness has been proved to associate with global cognitive status (Ascaso et al., 2014; Oktem et al., 2015) or episodic memory in MCI patients (Shen et al., 2014), as well as the progressive changes of cognition (Trebbastoni et al., 2016). Recently, two population-based cohort studies further reported that thinner RNFL was associated with current and future cognitive impairment, and could predict the incidence of dementia within 4.5 years in nondemented participants (Ko et al., 2018; Mutlu et al., 2018). These findings suggested that retinal and brain neurodegeneration may occur in parallel to some extent, and RNFL thickness could serve as a noninvasive and less expensive biomarker of AD.

Given the wide variance of RNFL thickness in old adults, OCT measurement at a single time point may not be informative enough to the identification of abnormal RNFL profiles in AD patients (Mok et al., 2002). In our previous studies, we found that longitudinal reduction of RNFL thickness was associated with the decline of episodic memory in old adults (Shen et al., 2013), and those who suffered cognitive decline within 25 months had more reduction of RNFL thickness with respect to old adults with stable cognition (Shi et al., 2014, 2016). It supports the hypothesis that a longitudinal change of RNFL thickness would be more indicative of cognitive deterioration among the elderly population.

However, whether the longitudinal changes of RNFL thickness associated with the classical AD biomarkers, such as atrophy of hippocampus, remains largely unknown at the present time. This gap of knowledge makes it difficult to ascertain whether the changes in RNFL thickness could reflect the pathologies of central nervous system. Therefore, we conducted a 12-month prospective cohort investigation as a pilot study to establish a system to validate the longitudinal association between RNFL thinning and specific brain atrophy in nondemented old adults. Our primary hypothesis was that RNFL attenuation was associated with hippocampus atrophy in older adults with normal cognition.

## Materials and Methods

### Study Population

This study protocol was approved by the Human Research Ethics Committee of the Tenth People’s Hospital in Shanghai, China (RES-2013015). Participants were recruited from the “Cognitive Training Program” *via* a flyer distribution (Cheng et al., 2012). All participants signed the informed consent form prior to being enrolled in the present study.

Totally, 50 community-based elderly adults with normal cognitive function and independent live capacity were eligible for screening from October 2012 to May 2013. The eligibility criteria included: (1) age 65 years or older; (2) 5 years or more of education and the Chinese version of Mini-Mental State Examination (CMMSE) score ≥ 24 points; (3) Chinese mandarin as their native language; and (4) having no disability and no difficulty in hearing, vision or communication. Exclusion criteria were: (1) prior diagnoses of neurologic diseases, e.g., dementia, Parkinson’s diseases, multiple sclerosis or stroke according to International Statistical Classification of Diseases and Related Health Problems 10th Revision (ICD-10; ICD-10-version, 2016); (2) history of mental disorders (e.g., major depressive disorder and schizophrenia) diagnosed according to the Diagnostic and Statistical Manual of Mental Disorders, 4th edition (DSM-IV; American-Psychiatric-Association, 1997); (3) known diseases contributing to the retina pathologies, e.g., diabetes, glaucoma or increased intraocular pressure (more than 22 mmHg), cataract and macular degeneration; and (4) having contraindications for MRI examination, e.g., metal implants, artificial joints or heart pacemaker.

Screening assessments for each participant were performed by research assistants and confirmed by professional psychiatrists. Demographic characteristics were collected including age, gender, education, height and weight. Moreover, the participants were arranged to have physical examinations and to check the basic laboratory results, such as liver function, renal function, and the levels of blood glucose and blood fats.

### Optical Coherence Tomography (OCT)

Retinal imaging was conducted on the same day as the cognitive assessment. The technologist performed all of the OCT examinations according to a standard protocol, and a board-certified ophthalmologist and retina specialist reviewed all of the OCT images to ensure the exclusion of participants with poor quality OCT images. Specifically, all the OCT scans were performed in a dark and enclosed room according to a repeat-scan protocol, which has been proved to be a precise method for measuring RNFL thickness in normal eyes (Cheung et al., 2008). HD-OCT 4000 (Carl Zeiss Meditec, Inc., Dublin, CA, USA) and intraocular pressure measurements (TX-20 Full Auto Tonometer; Canon 188 USA Inc., Irvine, CA, USA) were performed for all participants as described by Cheung et al. (2008). The polarization and Z-offset, as determined from the OCT settings, were optimized to assure the best possible scanning quality before the scan was obtained. A default Optic Disc Cube 200 × 200 protocol (software version 4.6) was used to determine the peripapillary RNFL thickness.

A scan was saved only if the fundus image was sufficiently visible to distinguish the optic disc and the scanning circle and if there were no obvious movement artifacts with missing data at the acquired scan pattern. Images with eye movements during scans, poor focus, or signal strength less than 4/10 were excluded. RNFL thickness was measured three times per eye and the average of these three measurements was taken for each eye. The RNFL thickness in superior, inferior, nasal and temporal quadrants were also recorded for each participant.

### Magnetic Resonance Imaging (MRI)

Structural MRI of the whole brain was acquired on a single 3T Gyroscan Intera system (Verio, Siemens, Erlangen, Germany) Scanner at baseline and after the follow-up 12 months. Images were obtained by using a standard 8-channel phased-array head coil. The head was stabilized with small cushions to minimize motion during scanning. A 3D anatomical T1-weighted dataset of the whole head was acquired using a 3D magnetization-prepared rapid acquisition gradient-echo (MPRAGE) sequence: repetition time (TR) = 1,900 ms, echo time (TE) = 3.43 ms, flip angle = 90°, field of view (FOV) = 256 mm, section thickness = 3 mm, matrix = 256 × 256, yielding 160 continuous sagittal slices of 1 mm thickness. MRI processing steps were performed by a research technician (LJ) who was blinded to all clinical information.

Voxel-based morphometry (VBM), within the statistical parametric mapping 8 (SPM8) was used to evaluate brain morphometry on a voxel-wise base. Customized templates and prior probability maps were created in order to reduce any potential normalization bias. Separate customized templates and priors were created for the baseline vs. follow-up comparisons. Therefore, in each comparison, all subjects were registered to the Montreal Neurological Institute (MNI) template using a 12° of freedom affine transformation and segmented into gray matter, white matter and CSF using MNI priors. Gray matter images were normalized to the MNI gray matter prior using a nonlinear discrete cosine transformation. The normalization parameters were applied to the original whole head and the images were segmented using the MNI priors. Average images were created of the whole head, gray matter, white matter and CSF, and smoothed using 8 mm full-width at half-maximum (FWHM) smoothing kernel.

FreeSurfer software package 5.3.0^1^ for Mac OS was used for automatic sub-cortical segmentation and volume estimates of T1-weighted images (Reuter et al., 2012). Several processing steps, such as skull stripping, Talairach transforms, atlas registration and spherical surface maps and parcellations were initialized with common information from the within-subject template, which significantly increased reliability and statistical power (Reuter et al., 2012). On the basis of the longitudinal processed images, the volumetric measurements of brain structures were performed by longitudinal pipeline of FreeSurfer software. All two-time points were first processed with the cross-sectional stream. Then, a base template was created from all two-time points, which operated as an initial estimate for the segmentation and surface reconstruction. The two measurement time points were then registered to this template to ensure non-biased analysis with regard to the two-time points. All reconstructed data were visually checked for segmentation accuracy at the baseline and follow-up. The MRI parameters included cortical gray matter volume, cortical white matter volume and white matter hyperintensity (WMH), as well as the volumes of specific brain structures, e.g., hippocampus and cingulate cortex.

### Neuropsychological Assessments

The general cognitive function at the enrollment was screened by using the CMMSE (Li et al., 2006) and the daily living function was assessed by using the Chinese version of Activities of Daily Living Scale (ADL; Chen et al., 1995), which included a Physical Self-Maintenance Scale (PSMS; six items) and an instrumental ADL (IADL; eight items). The Chinese version of Repeatable Battery for the Assessment of Neuropsychological Status (RBANS) was administrated to evaluate five specific cognitive domains for each participant: Immediate Memory, Visuospatial Memory, Language, Attention and Delayed Memory. We specifically focused on the cognitive domains of Immediate Memory (including subtests of list learning and story memory) and Delayed Memory (including subtests of list recall, list recognition, story recall and figure recall) in this study. The reliability and validity of RBANS in Chinese elderly community have been validated, and widely used in the field of neuropsychology (Cheng et al., 2011). Moreover, these tests have been used in our previous studies and showed great sensitivity to the cognitive function most vulnerable to aging (Shen et al., 2013, 2014; Shi et al., 2014). All the neuropsychological assessments were conducted by two trained researchers in the field of psychiatry (ZS and XC) according to the standard manual.

### Statistical Analysis

The *Kolmogorov-Smirnov* test was used to test the normality of all variables. The between-group differences were compared using a Student’s *t*-test for continuous variables [mean ± standard deviation (SD)] or a *Chi-square* test for categorical variables [N (%)]. *Paired t*-test was used to assess the changes of OCT, MRI and cognitive variables between baseline and follow-up measurements. Given that the measurement units for RNFL (μm) and brain structures (mm^3^) were dissimilar, raw data of longitudinal changes of OCT, MRI and cognitive variables were transferred into the change rate per year (%) for further statistical analysis. We initially applied a *Spearman* correlation to initially screen for the potential associations among RNFL thickness, brain structure volumes and cognitive function. And then, we used a multiple linear regression to predict such associations after adjustment of age and total intracranial volume. All analyses were conducted using SPSS version 20.0 (SPSS Inc., Chicago, IL, USA) and Prism 6 software (La Jolla, CA, USA), with *P* < 0.05 as the significance level.

## Results

### Characteristics of Participants

**Figure 1:**
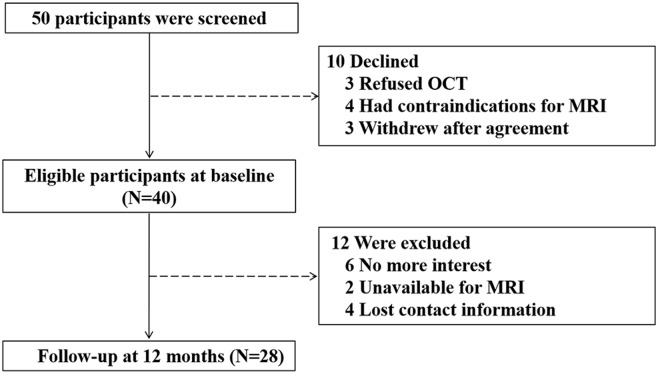
Flow diagram. The diagram shows that 50 participants were initially screened for the study. There were 40 participants eligible for baseline assessments and 28 participants completed the follow-up assessments after a period of 12 months. Finally, the data obtained from these 28 participants were included for the data analysis.

Fifty participants were screened and 10 of them were excluded: three refused to have OCT examination, four had contraindications to receive MRI scanning, and three withdrew from the study after signing the informed consent form. Thus, 40 participants were enrolled for the baseline study. The mean age of participants was 71.8 ± 3.9 years old, with 24 (60%) being male. The average CMMSE score was 27.0 ± 2.6 out of 30 points. After 12 months, 12 (30%) participants dropped from the study: six of them expressed a lack of interest in participating in the study, two of them were unavailable for MRI examination, and four of them lost the follow-up contact information. Finally, 28 participants were included in the data analysis (Figure 1). The demographic and clinical data of the participants were presented in Table 1. There were no significant differences in the baseline demographic or clinical features between the participants who stayed in the current studies and those who had dropped out from the studies.

**Table 1:** Baseline characteristics of the study population.

Variables	Total (*N* = 40)	Followed (*N* = 28)	Dropped out (*N* = 12)	*P*-value
Age (years)	71.8 ± 3.9	72.2 ± 4.0	70.9 ± 4.6	0.356
Gender, male, No. (%)	24 (60%)	14 (50%)	10 (83%)	0.105
Education (years)	10.2 ± 4.2	9.8 ± 4.3	11.1 ± 3.7	0.358
Height (cm)	159.2 ± 8.2	158.5 ± 6.7	160.8 ± 11.1	0.548
Weight (kg)	57.8 ± 13.5	59.1 ± 10.9	54.8 ± 18.5	0.353
IOP (mmHg)	16.6 ± 2.3	16.8 ± 2.2	16.3 ± 2.4	0.534
ADL score (point)	14.2 ± 0.9	14.2 ± 0.7	14.2 ± 0.7	0.339
CMMSE score (points)	27.0 ± 2.6	27.2 ± 2.3	26.5 ± 3.3	0.432
ICV (mm^3^)	1549670.4 ± 156034.0	1533584.0 ± 159324.0	1587205.2 ± 147708.2	0.326

*Abbreviation: IOP, intraocular pressure; ADL, Activities of Daily Living Scale; CMMSE, Chinese version of Mini-Mental State Examination; ICV, total intracranial volume. *P* values were calculated from *Studentt*-test or *Chi-square* test to compare the differences of baseline characteristics between the participants who stayed in the studies and the participants who dropped from the studies*.

### Longitudinal Changes of RNFL Thickness, Brain Structure Volume and Cognitive Function

First, we found that the changes in RNFL thickness within 12 months differed in four quadrants. Specifically, RNFL thickness in the superior quadrant was significantly thinner than that at the baseline examination (107.4 ± 13.9 μm vs. 102.8 ± 15.9 μm, *P* = 0.003), with a decreasing rate of 4.4%. While RNFL thickness in other quadrants did not present significant changes as compared to the baseline measurements. Moreover, the changes of different brain structure volumes showed a consistent decrease trend over the 12 months. The volumes of cortical gray matter (564886.3 ± 41772.6 mm^3^ vs. 561456.7 ± 40820.9 mm^3^, *P* < 0.001) and white matter (407043.0 ± 39187.4 mm^3^ vs. 402466.0 ± 38485.0 mm^3^, *P* < 0.001) reduced significantly as compared to the volumes of baseline assessments. The WMH increased by 13.6% (3644.1 ± 3094.8 mm^3^ vs. 4140.5 ± 3529.0 mm^3^, *P* < 0.001) during the period of 12-month follow-up. Furthermore, the volumes of hippocampus (7596.1 ± 900.5 mm^3^ vs. 7442.6 ± 883.1 mm^3^, *P* < 0.001) and the total cingulated cortex (2775.2 ± 334.1 mm^3^ vs. 2726.3 ± 332.3 mm^3^, *P* < 0.001) also demonstrated an obviously decreasing trend within 12 months. The longitudinal changes of other brain structure volumes, e.g., amygdale or caudate, were not statistically significant. As for the decline of specific cognitive domains measured by RBANS, we found the list learning (−12.0%, *P* < 0.001) and figure recall (−21.9%, *P* = 0.003) decreased significantly as compared to those at the baseline assessments. Moreover, the cognitive domains of Immediate Memory (−6.3%, *P* = 0.004) and Delayed Memory (−6.2%, *P* = 0.007) also reduced significantly from the baseline assessments over the follow-up 12 months (Table 2).

**Table 2:** Longitudinal changes of RNFL thickness, brain structure volume and cognitive function over 12 months.

Variables (*N* = 28)	Baseline	Follow-up	Change rate	*P*-value
**RNFL thickness**				
Average RNFL (μm)	87.6 ± 10.2	87.0 ± 8.5	−0.3%	<0.587
Superior RNFL (μm)	107.4 ± 13.9	102.8 ± 15.9	−4.4%	**<0.003**
Inferior RNFL (μm)	113.6 ± 19.5	111.2 ± 17.3	−1.4%	<0.155
Nasal RNFL (μm)	65.0 ± 9.0	65.3 ± 10.5	0.8%	<0.830
Temporal RNFL (μm)	65.2 ± 9.8	66.0 ± 9.6	2.2%	<0.624
**Brian structure volume**				
Gray matter (mm^3^)	564886.3 ± 41772.6	561456.7 ± 40820.9	−0.6%	**<0.001**
White matter (mm^3^)	407043.0 ± 39187.4	402466.0 ± 38485.0	−1.1%	**<0.001**
WMH (mm^3^)	3644.1 ± 3094.8	4140.5 ± 3529.0	13.6%	**<0.001**
Hippocampus (mm^3^)	7596.1 ± 900.5	7442.6 ± 883.1	−2.0%	**<0.001**
CC_mid_post (mm^3^)	325.4 ± 66.5	317.4 ± 61.6	−2.5%	**<0.004**
CC_anterior (mm^3^)	793.6 ± 142.7	773.0 ± 140.5	−2.6%	**<0.001**
CC_central (mm^3^)	373.7 ± 51.6	366.7 ± 50.9	−1.9%	**<0.047**
CC_total (mm^3^)	2775.2 ± 334.1	2726.3 ± 332.3	−1.8%	**<0.001**
**Cognitive function**				
List learning	28.8 ± 6.5	25.4 ± 7.6	−12.0%	**<0.001**
List recall	6.8 ± 3.0	5.8 ± 3.3	−15.3%	<0.087
List recognition	19.8 ± 0.5	19.5 ± 0.7	−1.2%	<0.070
Story memory	16.9 ± 3.6	16.3 ± 4.2	−3.8%	<0.236
Story recall	9.3 ± 2.0	8.5 ± 2.8	−8.5%	<0.102
Figure recall	16.1 ± 3.4	12.1 ± 5.8	−21.9%	**<0.003**
Immediate Memory	104.3 ± 16.5	97.8 ± 21.3	−6.3%	**<0.004**
Delayed Memory	113.5 ± 13.1	106.5 ± 14.1	−6.2%	**<0.007**

*Abbreviations: RNFL, retinal nerve fiber layer; WMH, white matter hyperintensities; CC, Cingulate cortex volume; CC_mid_post, middle posterior cingulate cortex volume; CC_anterior, anterior cingulated cortex volume; CC_central, central cingulated cortex volume; CC_total, total cingulated cortex volume. Change rates were defined by the ratio of the change value with the baseline value. *P* values were calculated from *Pairedt*-test to compare the RNFL thickness, brain structure volume and cognitive function between the baseline and the time of follow-up. Significant *P* values are indicated in bold*.

Giving that the aging process may differ in male and female subjects, we performed further analysis to compare RNFL reduction, brain atrophy and decline of cognitive function of different sex group over 12 months. The reduction of RNFL thickness in the inferior quadrant tended to be more significant in male participants in relative to female participants (*P* = 0.057). Besides, male participants showed more decline in Delayed Memory as compared to female participants (*P* = 0.019; Supplementary Table S1).

### Association Between RNFL Thinning and Brain Atrophy

The associations between the RNFL thinning and brain atrophy during the 12 months follow-up were analyzed. Results suggested that the average RNFL thinning was associated with the reduction of hippocampus volume (*R* = 0.38, *P* = 0.048), but the association disappeared after age and total intracranial volume being adjusted (*β* = 0.33, *P* = 0.104). Interestingly, we found that the reduction of RNFL thickness was selectively associated with a volumetric decrease of the cingulate cortex. The reduction of RNFL thickness in the superior quadrant was associated with the volumetric decline of middle posterior cingulate cortex (*R* = 0.41, *P* = 0.028) and total cingulate cortex (*R* = 0.40, *P* = 0.035), but the significance disappeared after the adjustment of age and total intracranial volume. While the average RNFL thinning was inversely associated with the decrease of central cingulate cortex volume after the adjustment of age and total intracranial volume (*β* = −0.41, *P* = 0.039). Specifically, the reduction of RNFL thickness in the inferior, not other quadrants, was independently associated with the decline of central cingulate cortex volume after the adjustment (*β* = −0.52, *P* = 0.006; Table 3). These data suggested that the reduction of RNFL thickness might reflect the atrophy process of cingulate cortex over 12 months in nondemented population.

**Table 3:** Associations between RNFL thinning and brain atrophy.

Reduction of RNFL	Decrease of brain structure volume							
	Gray matter	White matter	WMH	Hippocampus	CC_mid_post	CC_anterior	CC_central	CC_total
Average RNFL	0.38 (0.053)	−0.17 (0.397)	−0.01 (0.989)	0.33 (0.104)	0.04 (0.851)	0.24 (0.243)	**−0.41 (0.039)**	0.09 (0.632)
Superior RNFL	−0.01 (0.959)	0.13 (0.513)	0.06 (0.760)	0.26 (0.197)	0.37 (0.052)	0.10 (0.642)	0.06 (0.764)	0.31 (0.111)
Inferior RNFL	0.05 (0.792)	−0.07 (0.735)	−0.07 (0.722)	0.03 (0.872)	−0.01 (0.961)	−0.07 (0.724)	**−0.52 (0.006)**	0.01 (0.978)
Nasal RNFL	0.03 (0.901)	−0.21 (0.305)	0.13 (0.535)	0.27 (0.185)	−0.22 (0.274)	−0.21 (0.297)	−0.33 (0.092)	−0.16 (0.428)
Temporal RNFL	0.27 (0.183)	−0.20 (0.323)	−0.02 (0.921)	0.18 (0.389)	−0.12 (0.532)	0.09 (0.671)	−0.17 (0.394)	0.15 (0.456)

*Abbreviations: RNFL, retinal nerve fiber layer; WMH, white matter hyperintensities; CC, Cingulate cortex; CC_mid_post, middle posterior cingulate cortex volume; CC_anterior, anterior cingulated cortex volume; CC_central, central cingulated cortex volume; CC_total, total cingulated cortex volume. The data were represented as standardized regression coefficient (*P* values), which were generated with multivariate linear regression after the adjustment of age and total intracranial volume. Significant results are indicated in bold*.

### Association of RNFL Thinning and Brain Atrophy With Cognitive Decline

We determined the association between the changes of RNFL thickness and cognitive function during the follow-up period. We found that less reduction of average RNFL thickness was associated with a worse decrease in the test score of list recall (*β* = −0.48, *P* = 0.019) and the index score of Delayed Memory (*β* = −0.44, *P* = 0.017) after adjusting for age, gender and education years. Specifically, the reduction of RNFL thickness in the inferior quadrant (*β* = −0.49, *P* = 0.011) and temporal quadrant (*β* = −0.45, *P* = 0.039) were inversely associated with the decline of Delayed Memory after age, gender and education years being adjusted (Supplementary Table S2).

The association between the longitudinal changes of brain structure volume and cognitive function were analyzed at the same time. We found that the atrophy of anterior cingulate cortex (*β* = 0.45, *P* = 0.024) and mid-posterior cingulate cortex (*β* = 0.64, *P* = 0.001) were respectively associated with the decline in the test score of list recognition after adjusting for age, gender and education years. Moreover, the atrophy of mid-posterior cingulate cortex was associated with the decline in Delayed Memory (*β* = 0.45, *P* = 0.012) after the adjustment. In addition, we found that the aggregation of white matter hyperintensities was inversely associated with the decline in the test score of list recall (*β* = −0.42, *P* = 0.040) and list recognition (*β* = −0.48, *P* = 0.015) after adjusting for age, gender and education years (Supplementary Table S2).

In addition, we further analyzed the association of cognitive decline with RNFL thinning in *left* and *right* eye, brain atrophy in* left* and *right* hemisphere, respectively. We found that the reduction of average RNFL thickness in the *left* eye was inversely associated with Delayed Memory (*β* = −0.40, *P* = 0.043). The reduction of nasal RNFL thickness in the *right* eye was inversely associated with list learning (*β* = −0.44, *P* = 0.026) and list recognition (*β* = −0.41, *P* = 0.036). Moreover, the reduction of *right* hippocampus volume was inversely associated with the decline of test score of list learning (*β* = −0.52, *P* = 0.010) and decline of Immediate Memory (*β* = −0.53, *P* = 0.008; Supplementary Tables S3, S4).

Collectively, these findings suggested that RNFL thickness reduction was associated with cingulate cortex atrophy and cognitive deterioration, especially the decline of episodic memory over 12 months.

## Discussion

In this prospective study, we investigated the association between the longitudinal changes of RNFL thickness and brain structure volume in older adults with normal cognitive function within 12 months. We found that RNFL thinning was associated with the specific brain atrophy (e.g., central cingulate cortex) and the decline of episodic memory. The current results demonstrated the potential retinal-brain associations, which further validate RNFL thickness determined by OCT might be an inexpensive, noninvasive and easily reproducible biomarker for early detection and longitudinal monitoring of neurocognitive aging or AD.

A depletion of optic nerve ganglion cells and their axons might be part of the neurodegeneration and retinal measures were suggested to reflect global central nervous system pathology of neurodegeneration (London et al., 2013). Ocular manifestations in AD have been studied extensively over the few decades (Blanks et al., 1996; Kromer et al., 2014; Loh et al., 2017). As coalesce of axons of ganglion cells, RNFL thickness is originally supposed to provide a snapshot of the anterior visual pathway. But later studies found that ocular degeneration in AD was most severe at the posterior parts of the optic nerve (close to the optic chiasm), suggesting the initial site of ocular pathology was intracranial (Sadun and Bassi, 1990). Thus, RNFL thickness determined by OCT may represent the neurodegeneration of the brain. However, longitudinal change of RNFL thickness and the association of RNFL reduction and brain atrophy remain unclear.

In this study, RNFL thickness in the superior and inferior quadrants receded more significantly than the RNFL thickness in the other quadrants over the period of follow-up. Specifically, RNFL thickness in the inferior quadrant could be more affected in male participants. Previous studies have consistently reported the quadrant variations of RNFL attenuation in the aged population. Most studies suggested that the superior and inferior quadrants were more noticeably affected during the disease progression (Kirbas et al., 2013a; Liu et al., 2015); while some other studies showed a significant decrease of RNFL thickness in the temporal quadrant (Zhu et al., 2014; Wu et al., 2018). The exact reason why there is a regional variation of RNFL thickness reduction is not known at the present time. It could be due to the fact that the size of axons of RNFL degenerated differently in different quadrants of RNFL (Gelfand et al., 2012). Specifically, Gelfand et al. (2012) noted that affected areas of the retina contained axons derived from small parvocellular neurons that have small diameter axons. As is known, thinner axons are more vulnerable to insult that involves oxidative stress and aggregation of amyloid proteins during aging process (London et al., 2013). Future large-scale studies are warranted to observe the longitudinally changing of RNFL thickness in each quadrant across normal aging and patients with MCI or AD.

As for the longitudinal changes of brain structure volumes, we found a significant decrease of cortical gray and white matter volumes, hippocampus volume and cingulate cortex volume, and an apparent aggravation of white matter hyperintensities. However, there was no significant difference in brain atrophy between male and female individuals. These findings might also be due to the short period follow-up and small sample size. In the present study, it was originally expected that reduction of RNFL thickness would associate with hippocampus atrophy, the gold-standard biomarker of AD (Jack et al., 2011). Moreover, as a coalesce of axons of ganglion cells, RNFL thinning was expected to be associated with intracranial white matter atrophy or WMH aggravation. Unexpectedly, the reduction of average RNFL thickness was not associated with hippocampus atrophy, intracranial white matter atrophy or matter hyperintensity aggravation. Instead, we found that RNFL thinning was inversely associated with the decrease in cingulate cortex volume, which indicated that the less thinning rate of RNFL might mirror the greater intracranial atrophy of aging-sensitive brain structures, e.g., cingulate cortex.

These results confirmed the biological association between RNFL and intracranial structures and supported that the attenuation of RNFL could be an applaudable peripheral marker for observing intracranial neurodegeneration. However, the mechanisms underlying the inverse association remained unclear. Since previous evidences have suggested that the atrophy was not uniform across different brain regions, nor did it follow a linear trajectory (McDonald et al., 2009), and the reduction rate of RNFL thickness might not be linear patterns or paralleled with the atrophy rate of cingulate cortex volume, further studies with more time points will be necessary to investigate nonlinear effects of the neurodegenerative process (Sabuncu et al., 2011).

Although we have adjusted gender as a confounder when investigating the association between cognitive decline and RNFL or brain structures, we want to assess whether there is a difference in male and female participants. In the present study, female participants showed less decline in Delayed Memory within 12 months as compared to male participants. These results are supported by previous studies which emphasize the importance of sex differences in related to pathogenesis and prevention of AD [reviewed in Li and Singh (2014); Nebel et al. (2018)]. Consistently, Gerstorf et al. (2006) have reported that healthy elderly women usually always outperformed age-matched men on episodic memory tasks.

Moreover, we found that the reduction of RNFL thickness was inversely associated with the decline of episodic memory (e.g., list recall and delayed memory) after adjusted age, gender and education, namely, the less but not greater annual reduction rate of RNFL thickness indicated severe cognitive decline. These findings were consistent with our previous studies (Shen et al., 2013; Shi et al., 2014), which might be due to the gliotic reactive changes in the pathogenesis of inner retina. Specifically, gliosis, hypertrophy and proliferation of astrocytes and astrocytic neuroinflammation may occur before the onset of neuronal death and therefore may obfuscate the detection of thinner RNFL (Knoll et al., 2016).

Meanwhile, white matter hyperintensities aggravation and the cingulate cortex atrophy were also associated with the decline of episodic memory after the adjustment of age, gender and education. Accordingly, a higher global white matter hyperintensities burden appeared to increase the risk of cognitive decline in elderly adults (Nolze-Charron et al., 2015). These results lend support to the previous findings that patients with white matter hyperintensities are at an increased risk of clinical progression and cognitive decline, and are vulnerable to suffer the deficits of episodic memory (Fujishima et al., 2014; Benedictus et al., 2015). The possible explanation was that white matter hyperintensities disrupt the cholinergic pathway and contribute to the deterioration of cognitive function (Bocti et al., 2005). In addition, we also demonstrated that cingulate cortex could be more vulnerable to the age-related neurodegeneration than the other brain regions in cognitively normal older adults, which was consistent with previous findings (Mann et al., 2011). Cingulate cortex is the central hub in cognitive brain networks (Jockwitz et al., 2016). Particularly, posterior cingulate cortex has important relevance to process episodic memory tasks (Brugnolo et al., 2014). Atrophy of posterior cingulate cortex could characterize old individuals at risk of developing AD (Chételat et al., 2005; Conway et al., 2006; Teipel and Grothe, 2016). The findings from current studies further suggest that cingulate cortex plays a key role in neurodegeneration during the process of aging. Additionally, we found that hippocampus atrophy in the *right*, not the *left*, hemisphere was inversely associated with the cognitive decline of Immediate Memory (determined by worst list learning test). We speculated that the discrepancy between* left* and *right* hemisphere might be attributed to the compensatory mechanism of non-dominant hemisphere, as suggested in previous studies (Huijbers et al., 2015).

There were several limitations to the current study. First, the sample size at the follow-up assessment was small (*N* = 28), and the drop-off rate was relatively high (30%). However, we compared the demographic characteristics of the participants who stayed for the study and the participants who dropped from the study, and we did not find any significant differences between the two populations (Table 1). Second, we did not adjust for multiple comparisons when calculating the retinal-brain associations, thus the identified association and the statistical power should be confirmed in a larger scale cohort. Finally, the current study only included cognitively normal older adults and the follow-up period of 12 months was relatively short to observe the cognitive conversion in the participants with normal cognitive function. It remains unclear whether these findings could be repeated in both MCI and AD dementia patients. But serving as a pilot investigation, we established a system to further validate the potential value of RNFL thickness reduction as a biomarker of AD.

In conclusion, longitudinal reduction of RNFL thickness measured by OCT could reflect the intracranial neurodegenerative process and cognitive impairment in aging participants. OCT measurement, a noninvasive, reproducible, rapid and well-tolerated examination for retinal characteristics, would be a useful complementary technique to MRI in evaluating longitudinal changes of neurodegeneration. It provides the advantages of detecting participants at the prodromal stage of AD and targeting population for cognitive intervention.

## Data Availability

All datasets generated for this study are included in the manuscript and/or the Supplementary Files.

## Author Contributions

YS and CL: study concept and design, administrative, technical, material support and study supervision. ZS, HZ, JH, LJ, XC, YC, XM, CL and YS: study conduct, acquisition, analysis and interpretation of data. ZS and YS: drafting of the manuscript. ZS, CL and YS: critically revising article for important intellectual content. ZS, HZ, JH, LJ, XC, YC, XM, CL and YS: final approval of the version to be published, agreement to be accountable for the work in ensuring that related questions are appropriately investigated and resolved.

## Conflict of Interest Statement

The authors declare that the research was conducted in the absence of any commercial or financial relationships that could be construed as a potential conflict of interest.

## Footnotes

^1^http://surfer.nmr.mgh.harvard.edu

## References

[B1] American-Psychiatric-Association (1997). Diagnostic and Statistical Manual of Mental Disorders. 4th Edn. Washington, DC: American Psychiatric Association.

[B2] Ascaso F. J. Cruz N. Modrego P. J. Lopez-Anton R. Santabárbara J. Pascual L. F.. (2014). Retinal alterations in mild cognitive impairment and Alzheimer’s disease: an optical coherence tomography study. J. Neurol. 261, 1522–1530. 10.1007/s00415-014-7374-z 24846203

[B3] Bateman R. J. Xiong C. Benzinger T. L. S. Fagan A. M. Goate A. Fox N. C.. (2012). Clinical and biomarker changes in dominantly inherited Alzheimer’s disease. N. Engl. J. Med. 367, 795–804. 10.1056/NEJMoa1202753 22784036 PMC3474597

[B4] Benedictus M. R. van Harten A. C. Leeuwis A. E. Koene T. Scheltens P. Barkhof F.. (2015). White matter hyperintensities relate to clinical progression in subjective cognitive decline. Stroke 46, 2661–2664. 10.1161/strokeaha.115.009475 26173729

[B5] Blanks J. C. Schmidt S. Y. Torigoe Y. Porrello K. V. Hinton D. R. Blanks R. H. (1996). Retinal pathology in Alzheimer’s disease. II. Regional neuron loss and glial changes in GCL. Neurobiol. Aging 17, 385–395. 10.1016/0197-4580(96)00009-7 8725900

[B6] Bocti C. Swartz R. H. Gao F. Q. Sahlas D. J. Behl P. Black S. E. (2005). A new visual rating scale to assess strategic white matter hyperintensities within cholinergic pathways in dementia. Stroke 36, 2126–2131. 10.1161/01.str.0000183615.07936.b6 16179569

[B7] Brugnolo A. Morbelli S. Arnaldi D. De Carli F. Accardo J. Bossert I.. (2014). Metabolic correlates of Rey auditory verbal learning test in elderly subjects with memory complaints. J. Alzheimers Dis. 39, 103–113. 10.3233/jad-121684 24150105

[B8] Bussel I. I. Wollstein G. Schuman J. S. (2014). OCT for glaucoma diagnosis, screening and detection of glaucoma progression. Br. J. Ophthalmol. 98, ii15–ii19. 10.1136/bjophthalmol-2013-304326 24357497 PMC4208340

[B9] Chen P. Yu E. S. Zhang M. Liu W. T. Hill R. Katzman R. (1995). ADL dependence and medical conditions in Chinese older persons: a population-based survey in Shanghai, China. J. Am. Geriatr. Soc. 43, 378–383. 10.1111/j.1532-5415.1995.tb05811.x 7706627

[B10] Cheng Y. Wu W. Feng W. Wang J. Chen Y. Shen Y.. (2012). The effects of multi-domain versus single-domain cognitive training in non-demented older people: a randomized controlled trial. BMC Med. 10:30. 10.1186/1741-7015-10-30 22453114 PMC3364144

[B11] Cheng Y. Wu W. Wang J. Feng W. Wu X. Li C. (2011). Reliability and validity of the repeatable battery for the assessment of neuropsychological status in community-dwelling elderly. Arch. Med. Sci. 7, 850–857. 10.5114/aoms.2011.25561 22291831 PMC3258798

[B12] Chételat G. Landeau B. Eustache F. Mézenge F. Viader F. de la Sayette V.. (2005). Using voxel-based morphometry to map the structural changes associated with rapid conversion in MCI: a longitudinal MRI study. Neuroimage 27, 934–946. 10.1016/j.neuroimage.2005.05.015 15979341

[B13] Cheung C. Y. Leung C. K. Lin D. Pang C. P. Lam D. S. (2008). Relationship between retinal nerve fiber layer measurement and signal strength in optical coherence tomography. Ophthalmology 115, 1347–1351. 10.1016/j.ophtha.2007.11.027 18294689

[B14] Conway C. R. Sheline Y. I. Chibnall J. T. George M. S. Fletcher J. W. Mintun M. A. (2006). Cerebral blood flow changes during vagus nerve stimulation for depression. Psychiatry Res. 146, 179–184. 10.1016/j.pscychresns.2005.12.007 16510266

[B15] den Haan J. Verbraak F. D. Visser P. J. Bouwman F. H. (2017). Retinal thickness in Alzheimer’s disease: a systematic review and meta-analysis. Alzheimers Dement. 6, 162–170. 10.1016/j.dadm.2016.12.014 28275698 PMC5328759

[B16] Doustar J. Torbati T. Black K. L. Koronyo Y. Koronyo-Hamaoui M. (2017). Optical coherence tomography in Alzheimer’s disease and other neurodegenerative diseases. Front. Neurol. 8:701. 10.3389/fneur.2017.00701 29312125 PMC5742098

[B17] Fujishima M. Maikusa N. Nakamura K. Nakatsuka M. Matsuda H. Meguro K. (2014). Mild cognitive impairment, poor episodic memory, and late-life depression are associated with cerebral cortical thinning and increased white matter hyperintensities. Front. Aging Neurosci. 6:306. 10.3389/fnagi.2014.00306 25426066 PMC4224123

[B18] Gelfand J. M. Goodin D. S. Boscardin W. J. Nolan R. Cuneo A. Green A. J. (2012). Retinal axonal loss begins early in the course of multiple sclerosis and is similar between progressive phenotypes. PLoS One 7:e36847. 10.1371/journal.pone.0036847 22666330 PMC3359324

[B19] Gerstorf D. Herlitz A. Smith J. (2006). Stability of sex differences in cognition in advanced old age: the role of education and attrition. J. Gerontol. B Psychol. Sci. Soc. Sci. 61, P245–P249. 10.1093/geronb/61.4.p245 16855037

[B20] Hampel H. Bürger K. Teipel S. J. Bokde A. L. Zetterberg H. Blennow K. (2008). Core candidate neurochemical and imaging biomarkers of Alzheimer’s disease. Alzheimers Dement. 4, 38–48. 10.1016/j.jalz.2007.08.006 18631949

[B21] He X. F. Liu Y. T. Peng C. Zhang F. Zhuang S. Zhang J. S. (2012). Optical coherence tomography assessed retinal nerve fiber layer thickness in patients with Alzheimer’s disease: a meta-analysis. Int. J. Ophthalmol. 5, 401–405. 10.3980/j.issn.2222-3959.2012.03.30 22773997 PMC3388417

[B22] Hinton D. R. Sadun A. A. Blanks J. C. Miller C. A. (1986). Optic-nerve degeneration in Alzheimer’s disease. N. Engl. J. Med. 315, 485–487. 10.1056/NEJM198608213150804 3736630

[B23] Huang D. Swanson E. Lin C. Schuman J. Stinson W. Chang W.. (1991). Optical coherence tomography. Science 254, 1178–1181. 10.1126/science.1957169 1957169 PMC4638169

[B24] Huijbers W. Mormino E. Schultz A. Wigman S. Ward A. M. Larvie M.. (2015). Amyloid-β deposition in mild cognitive impairment is associated with increased hippocampal activity, atrophy and clinical progression. Brain 138, 1023–1035. 10.1093/brain/awv007 25678559 PMC4438387

[B25] ICD-10-version (2016). Available online at: http://apps.who.int/classifications/icd10/browse/2016/en

[B26] Jack C. R. Jr. Albert M. S. Knopman D. S. McKhann G. M. Sperling R. A. Carrillo M. C.. (2011). Introduction to the recommendations from the National Institute on Aging-Alzheimer’s Association workgroups on diagnostic guidelines for Alzheimer’s disease. Alzheimers Dement. 7, 257–262. 10.1016/j.jalz.2011.03.004 21514247 PMC3096735

[B27] Jack C. R. Knopman D. S. Jagust W. J. Petersen R. C. Weiner M. W. Aisen P. S.. (2013). Tracking pathophysiological processes in Alzheimer’s disease: an updated hypothetical model of dynamic biomarkers. Lancet Neurol. 12, 207–216. 10.1016/s1474-4422(12)70291-0 23332364 PMC3622225

[B28] Jockwitz C. Caspers S. Lux S. Jutten K. Schleicher A. Eickhoff S. B.. (2016). Age- and function-related regional changes in cortical folding of the default mode network in older adults. Brain Struct. Funct. 222, 83–99. 10.1007/s00429-016-1202-4 26943919

[B29] Kirbas S. Turkyilmaz K. Anlar O. Tufekci A. Durmus M. (2013a). Retinal nerve fiber layer thickness in patients with Alzheimer disease. J. Neuroophthalmol. 33, 58–61. 10.1097/wno.0b013e318267fd5f 22918296

[B30] Kirbas S. Turkyilmaz K. Tufekci A. Durmus M. (2013b). Retinal nerve fiber layer thickness in Parkinson disease. J. Neuroophthalmol. 33, 62–65. 10.1097/wno.0b013e3182701745 23100041

[B31] Knoll B. Simonett J. Volpe N. J. Farsiu S. Ward M. Rademaker A.. (2016). Retinal nerve fiber layer thickness in amnestic mild cognitive impairment: case-control study and meta-analysis. Alzheimers Dement. 4, 85–93. 10.1016/j.dadm.2016.07.004 27722194 PMC5045947

[B32] Ko F. Muthy Z. A. Gallacher J. Sudlow C. Rees G. Yang Q.. (2018). Association of retinal nerve fiber layer thinning with current and future cognitive decline: a study using optical coherence tomography. JAMA Neurol. 75, 1198–1205. 10.1001/jamaneurol.2018.1578 29946685 PMC6233846

[B33] Kromer R. Serbecic N. Hausner L. Froelich L. Aboul-Enein F. Beutelspacher S. C. (2014). Detection of retinal nerve fiber layer defects in Alzheimer’s disease using SD-OCT. Front. Psychiatry 5:22. 10.3389/fpsyt.2014.00022 24616709 PMC3934110

[B35] Li R. Singh M. (2014). Sex differences in cognitive impairment and Alzheimer’s disease. Front. Neuroendocrinol. 35, 385–403. 10.1016/j.yfrne.2014.01.002 24434111 PMC4087048

[B34] Li C. Wu W. Jin H. Zhang X. Xue H. He Y.. (2006). Successful aging in Shanghai, China: definition, distribution and related factors. Int. Psychogeriatr. 18, 551–563. 10.1017/s1041610205002966 16478568

[B36] Liu D. Zhang L. Li Z. Zhang X. Wu Y. Yang H.. (2015). Thinner changes of the retinal nerve fiber layer in patients with mild cognitive impairment and Alzheimer’s disease. BMC Neurol. 15:14. 10.1186/s12883-015-0268-6 25886372 PMC4342899

[B37] Loh E. H.-T. Ong Y.-T. Venketasubramanian N. Hilal S. Thet N. Wong T. Y.. (2017). Repeatability and reproducibility of retinal neuronal and axonal measures on spectral-domain optical coherence tomography in patients with cognitive impairment. Front. Neurol. 8:359. 10.3389/fneur.2017.00359 28861029 PMC5559462

[B38] London A. Benhar I. Schwartz M. (2013). The retina as a window to the brain-from eye research to CNS disorders. Nat. Rev. Neurol. 9, 44–53. 10.1038/nrneurol.2012.227 23165340

[B39] Mann S. L. Hazlett E. A. Byne W. Hof P. R. Buchsbaum M. S. Cohen B. H.. (2011). Anterior and posterior cingulate cortex volume in healthy adults: effects of aging and gender differences. Brain Res. 1401, 18–29. 10.1016/j.brainres.2011.05.050 21669408 PMC3134959

[B40] McDonald C. R. McEvoy L. K. Gharapetian L. Fennema-Notestine C. Hagler D. J. Holland D.. (2009). Regional rates of neocortical atrophy from normal aging to early Alzheimer disease. Neurology 73, 457–465. 10.1212/wnl.0b013e3181b16431 19667321 PMC2727145

[B41] Mok K. H. Lee V. W. So K. F. (2002). Retinal nerve fiber layer measurement of the Hong Kong chinese population by optical coherence tomography. J. Glaucoma 11, 481–483. 10.1097/00061198-200212000-00004 12483090

[B42] Mutlu U. Colijn J. M. Ikram M. A. Bonnemaijer P. W. M. Licher S. Wolters F. J.. (2018). Association of retinal neurodegeneration on optical coherence tomography with dementia: a population-based study. JAMA Neurol. 75, 1256–1263. 10.1001/jamaneurol.2018.1563 29946702 PMC6233847

[B43] Nebel R. A. Aggarwal N. T. Barnes L. L. Gallagher A. Goldstein J. M. Kantarci K.. (2018). Understanding the impact of sex and gender in Alzheimer’s disease: a call to action. Alzheimers Dement. 14, 1171–1183. 10.1016/j.jalz.2018.04.008 29907423 PMC6400070

[B44] Nolze-Charron G. Mouiha A. Duchesne S. Bocti C. (2015). White matter hyperintensities in mild cognitive impairment and lower risk of cognitive decline. J. Alzheimers Dis. 46, 855–862. 10.3233/jad-140618 26402625

[B45] Oktem E. O. Derle E. Kibaroglu S. Oktem C. Akkoyun I. Can U. (2015). The relationship between the degree of cognitive impairment and retinal nerve fiber layer thickness. Neurol. Sci. 36, 1141–1146. 10.1007/s10072-014-2055-3 25575807

[B46] Price J. L. Morris J. C. (1999). Tangles and plaques in nondemented aging and “preclinical” Alzheimer’s disease. Ann. Neurol. 45, 358–368. 10.1002/1531-8249(199903)45:3<358::aid-ana12>3.0.co;2-x 10072051

[B47] Reuter M. Schmansky N. J. Rosas H. D. Fischl B. (2012). Within-subject template estimation for unbiased longitudinal image analysis. Neuroimage 61, 1402–1418. 10.1016/j.neuroimage.2012.02.084 22430496 PMC3389460

[B48] Sabuncu M. R. Desikan R. S. Sepulcre J. Yeo B. T. Liu H. Schmansky N. J.. (2011). The dynamics of cortical and hippocampal atrophy in alzheimer disease. Arch. Neurol. 68, 1040–1048. 10.1001/archneurol.2011.167 21825241 PMC3248949

[B49] Sadun A. A. Bassi C. J. (1990). Optic nerve damage in Alzheimer’s disease. Ophthalmology 97, 9–17. 10.1016/s0161-6420(90)32621-0 2314849

[B50] Saidha S. Sotirchos E. S. Oh J. Syc S. B. Seigo M. A. Shiee N.. (2013). Relationships between retinal axonal and neuronal measures and global central nervous system pathology in multiple sclerosis. JAMA Neurol. 70, 34–43. 10.1001/jamaneurol.2013.573 23318513 PMC4030557

[B51] Shen Y. Liu L. Cheng Y. Feng W. Shi Z. Zhu Y.. (2014). Retinal nerve fiber layer thickness is associated with episodic memory deficit in mild cognitive impairment patients. Curr. Alzheimer Res. 11, 259–266. 10.2174/1567205011666140131114418 24484274

[B52] Shen Y. Shi Z. Jia R. Zhu Y. Cheng Y. Feng W.. (2013). The attenuation of retinal nerve fiber layer thickness and cognitive deterioration. Front. Cell. Neurosci. 7:142. 10.3389/fncel.2013.00142 24065883 PMC3777215

[B53] Shi Z. Wu Y. Wang M. Cao J. Feng W. Cheng Y.. (2014). Greater attenuation of retinal nerve fiber layer thickness in Alzheimer’s disease patients. J. Alzheimers Dis. 40, 277–283. 10.3233/JAD-131898 24413621

[B54] Shi Z. Zhu Y. Wang M. Wu Y. Cao J. Li C.. (2016). The utilization of retinal nerve fiber layer thickness to predict cognitive deterioration. J. Alzheimers Dis. 49, 399–405. 10.3233/jad-150438 26484909

[B55] Soldan A. Pettigrew C. Cai Q. Wang M. C. Moghekar A. R. O’Brien R. J.. (2016). Hypothetical preclinical alzheimer disease groups and longitudinal cognitive change. JAMA Neurol. 73, 698–705. 10.1001/jamaneurol.2016.0194 27064267 PMC5173327

[B56] Sperling R. Mormino E. Johnson K. (2014). The evolution of preclinical Alzheimer’s disease: implications for prevention trials. Neuron 84, 608–622. 10.1016/j.neuron.2014.10.038 25442939 PMC4285623

[B57] Teipel S. Grothe M. J. (2016). Does posterior cingulate hypometabolism result from disconnection or local pathology across preclinical and clinical stages of Alzheimer’s disease? Eur. J. Nucl. Med. Mol. Imaging 43, 526–536. 10.1007/s00259-015-3222-3 26555082 PMC6166099

[B58] Thomson K. L. Yeo J. M. Waddell B. Cameron J. R. Pal S. (2015). A systematic review and meta-analysis of retinal nerve fiber layer change in dementia, using optical coherence tomography. Alzheimers Dement. Amst. 1, 136–143. 10.1016/j.dadm.2015.03.001 27239501 PMC4876885

[B59] Tosun D. Landau S. Aisen P. S. Petersen R. C. Mintun M. Jagust W.. (2017). Association between tau deposition and antecedent amyloid-β accumulation rates in normal and early symptomatic individuals. Brain 140, 1499–1512. 10.1093/brain/awx046 28334939

[B60] Trebbastoni A. D’Antonio F. Bruscolini A. Marcelli M. Cecere M. Campanelli A.. (2016). Retinal nerve fibre layer thickness changes in Alzheimer’s disease: results from a 12-month prospective case series. Neurosci. Lett. 629, 165–170. 10.1016/j.neulet.2016.07.006 27394689

[B61] Visser P. J. Verhey F. Knol D. L. Scheltens P. Wahlund L. O. Freund-Levi Y.. (2009). Prevalence and prognostic value of CSF markers of Alzheimer’s disease pathology in patients with subjective cognitive impairment or mild cognitive impairment in the DESCRIPA study: a prospective cohort study. Lancet Neurol. 8, 619–627. 10.1016/S1474-4422(09)70139-5 19523877

[B62] Wu Y. Wang X. N. Wang N. Han Y. Ma D. Lu Y. (2018). Regularity changes of the retinal nerve fiber layer and macular ganglion cell complex in patients with the amnestic mild cognitive impairment. Int. J. Neurosci. 128, 849–853. 10.1080/00207454.2018.1438428 29447481

[B63] Yaffe K. Tocco M. Petersen R. C. Sigler C. Burns L. C. Cornelius C.. (2012). The epidemiology of Alzheimer’s disease: laying the foundation for drug design, conduct, and analysis of clinical trials. Alzheimers Dement. 8, 237–242. 10.1016/j.jalz.2011.12.005 22546356

[B64] Zhu L. Ren X. Wang Y. Xu L. Zhang X. (2014). Retinal nerve fiber layer thickness in the patients with mild cognitive impairment or Alzheimer’s disease. Ophthalmol 23, 231–234. 10.13281/j.cnki.issn.1004-4469.2014.04.00430865872

