# Supplementary material for: Retinal Nerve Fiber Layer Thinning Is Associated With Brain Atrophy: A Longitudinal Study in Nondemented Older Adults
Source: Front Aging Neurosci. 2019 Apr 11;11:69. doi: 10.3389/fnagi.2019.00069 (PMC6470389; doi:10.3389/fnagi.2019.00069)
Supplement: Supplementary file 1 [file Table_1.docx]

**Supplementary Table S1. Comparison of RNFL thinning, brain atrophy and cognitive decline over 12 months between male and female participants**

| Variables (N=28) | Male (N=15) | Female (N=13) | *P* value |
| --- | --- | --- | --- |
| *RNFL thinning* | | | |
| Average RNFL (μm) | -1.6 ± 6.2 | 0.4 ± 6.3 | 0.406 |
| Superior RNFL (μm) | -6.0 ± 7.2 | -3.2 ± 8.1 | 0.345 |
| Inferior RNFL (μm) | -5.2 ± 7.1 | 0.9 ± 9.2 | 0.057 |
| Nasal RNFL (μm) | 0.4 ± 7.7 | 0.2 ± 6.2 | 0.949 |
| Temporal RNFL (μm) | -0.9 ± 3.7 | 2.8 ± 11.9 | 0.266 |
| *Brain atrophy* | | | |
| Grey matter (mm^3^) | -2673.2 ± 9201.5 | -1058.0 ± 5350.6 | 0.583 |
| White matter (mm^3^) | -3919.3 ± 5885.5 | -5335.8 ± 3783.5 | 0.451 |
| WMH (mm^3^) | 550.0 ± 771.2 | 434.6 ± 443.7 | 0.639 |
| Hippocampus (mm^3^) | -168.9 ± 153.8 | -135.7 ± 102.7 | 0.516 |
| CC_mid_post (mm^3^) | -10.3 ± 14.4 | -5.5 ± 13.0 | 0.369 |
| CC_anterior (mm^3^) | -17.4 ± 17.1 | -24.4 ± 25.6 | 0.399 |
| CC_central (mm^3^) | -9.1 ± 15.0 | -4.6 ± 20.9 | 0.514 |
| CC_total (mm^3^) | -56.7 ± 47.6 | -40.0 ± 59.2 | 0.416 |
| *Cognitive decline* | | | |
| List learning | -4.1 ± 5.1 | -2.8 ± 2.8 | 0.421 |
| List recall | -1.3 ± 3.1 | -0.8 ± 3.2 | 0.680 |
| List recognition | -0.4 ± 0.7 | -0.1 ± 0.6 | 0.230 |
| Story memory | -0.5 ± 2.8 | -0.8 ± 2.9 | 0.829 |
| Story recall | -1.1 ± 2.3 | -0.5 ± 2.7 | 0.526 |
| Figure recall | -4.8 ± 5.5 | -3.1 ± 7.4 | 0.488 |
| Immediate Memory | -6.3 ± 13.0 | -6.8 ± 8.1 | 0.918 |
| Delayed Memory | -12.2 ± 12.3 | -1.2 ± 10.7 | **0.019** |

Abbreviations: RNFL, retinal nerve fiber layer; WMH, white matter hyperintensities; CC, Cingulate cortex volume; CC_mid_post, middle posterior cingulate cortex volume; CC_anterior, anterior cingulated cortex volume; CC_central, central cingulated cortex volume; CC_total, total cingulated cortex volume. *P* values were calculated from *Student* *t*-test to compare the RNFL thinning, brain atrophy and cognitive decline over 12 months between male and female individuals. Significant *P* values are indicated in bold.

**Supplementary Table S2. Association of cognitive decline with RNFL thinning and brain atrophy**

|  | *Decline of cognitive function* | | | | | | | | |
| --- | --- | --- | --- | --- | --- | --- | --- | --- | --- |
|  | | List learning | List recall | List recognition | Story memory | Story recall | Figure recall | Immediate Memory | Delayed Memory |
| *Reduction of RNFL thickness* | | | | | | | | | |
| Average RNFL | | -0.09 (0.684) | **-0.48 (0.019)** | -0.04 (0.081) | 0.03 (0.903) | -0.21 (0.317) | 0.01 (0.962) | 0.04 (0.856) | **-0.44 (0.017)** |
| Superior RNFL | | -0.01(0.989) | -0.22 (0.298) | 0.14 (0.499) | 0.14 (0.903) | 0.21 (0.299) | 0.05 (0.815) | 0.16 (0.441) | 0.07 (0.711) |
| Inferior RNFL | | -0.01 (0.957) | -0.37 (0.093) | 0.10 (0.648) | -0.13 (0.546) | -0.36 (0.082) | 0.23 (0.307) | 0.06 (0.770) | **-0.49 (0.011)** |
| Nasal RNFL | | -0.17 (0.403) | -0.27 (0.196) | -0.22 (0.297) | 0.23 (0.241) | 0.20 (0.323) | -0.16 (0.451) | 0.11 (0.598) | -0.07 (0.703) |
| Temporal RNFL | | 0.05 (0.835) | -0.25 (0.322) | -0.23 (0.292) | 0.08 (0.741) | -0.38 (0.101) | 0.01 (0.955) | 0.12 (0.620) | **-0.45 (0.039)** |
| *Decrease of brain structure volume* | | | | | | | | | |
| Grey matter | | -0.17 (0.413) | -0.10 (0.624) | 0.12 (0.448) | -0.16 (0.428) | 0.15 (0.467) | -0.07 (0.736) | -0.30 (0.149) | 0.03 (0.885) |
| White matter | | 0.12 (0.586) | -0.14 (0.544) | 0.30 (0.148) | -0.17 (0.428) | -0.35 (0.102) | 0.35 (0.109) | -0.05 (0.814) | -0.15 (0.456) |
| WMH | | -0.29 (0.165) | **-0.42 (0.040)** | **-0.48 (0.015)** | -0.11 (0.578) | -0.28 (0.164) | 0.12 (0.576) | -0.33 (0.105) | -0.32 (0.090) |
| Hippocampus | | -0.28 (0.189) | -0.13 (0.540) | 0.28 (0.177) | -0.04 (0.832) | 0.27 (0.186) | -0.14 (0.512) | -0.24 (0.256) | 0.16 (0.424) |
| CC_mid_post | | -0.01 (0.989) | 0.14 (0.502) | **0.64 (0.001)** | -0.02 (0.907) | 0.29 (0.152) | 0.15 (0.470) | -0.07 (0.751) | **0.45 (0.012)** |
| CC_anterior | | 0.07 (0.756) | 0.12 (0.578) | **0.45 (0.024)** | 0.18 (0.372) | 0.04 (0.841) | -0.07 (0.725) | 0.06 (0.777) | 0.10 (0.600) |
| CC_total | | 0.11 (0.603) | -0.04 (0.849) | 0.07 (0.751) | 0.29 (0.161) | 0.28 (0.171) | -0.24 (0.249) | 0.20 (0.336) | 0.04 (0.858) |

Abbreviations: RNFL, retinal nerve fiber layer; WMH, white matter hyperintensities; CC, Cingulate cortex; CC_mid_post, middle posterior cingulate cortex volume; CC_anterior, anterior cingulated cortex volume; CC_total, total cingulated cortex volume. The data were represented as standardized regression coefficient (*P* values), which were generated with multivariable linear regression adjusting for age, gender and education years. Significant results are indicated in bold.

**Supplementary Table S3. Association of cognitive decline with RNFL thinning in *left* eye and brain atrophy in *left* hemisphere**

|  | *Decline of cognitive function* | | | | | | | | |
| --- | --- | --- | --- | --- | --- | --- | --- | --- | --- |
|  | | List learning | List recall | List recognition | Story memory | Story recall | Figure recall | Immediate Memory | Delayed Memory |
| *Reduction of RNFL thickness in left eye* | | | | | | | | | |
| Average RNFL | | 0.01 (0.991) | -0.38 (0.080) | -0.23 (0.298) | 0.06 (0.789) | -0.16 (0.457) | 0.12 (0.609) | 0.14 (0.529) | **-0.40 (0.043)** |
| Superior RNFL | | -0.03 (0.905) | -0.39 (0.072) | 0.03 (0.893) | 0.04 (0.848) | 0.14 (0.520) | 0.11 (0.614) | 0.09 (0.687) | -0.20 (0.306) |
| Inferior RNFL | | -0.02 (0.930) | -0.24 (0.262) | -0.13 (0.545) | -0.01 (0.981) | -0.24 (0.240) | 0.18 (0.408) | 0.07 (0.756) | -0.28 (0.141) |
| Nasal RNFL | | -0.02 (0.923) | -0.24 (0.273) | -0.20 (0.343) | 0.06 (0.775) | -0.08 (0.704) | 0.10 (0.649) | 0.13 (0.533) | -0.24 (0.234) |
| Temporal RNFL | | 0.09 (0.724) | -0.21 (0.398) | 0.38 (0.116) | 0.07 (0.762) | -0.30 (0.209) | 0.09 (0.715) | 0.11 (0.663) | -0.43 (0.053) |
| *Decrease of hippocampus volume in left hemisphere* | | | | | | | | | |
| Hippocampus | | 0.12 (0.568) | 0.04 (0.859) | 0.18 (0.361) | 0.11 (0.565) | 0.06 (0.784) | -0.09 (0.674) | 0.18 (0.371) | -0.01 (0.993) |

Abbreviations: RNFL, retinal nerve fiber layer. The data were represented as standardized regression coefficient (*P* values), which were generated with multivariable linear regression adjusting for age, gender and education years. Significant results are indicated in bold.

**Supplementary Table S4. Association of cognitive decline with RNFL thinning in *right* eye and brain atrophy in *right* hemisphere**

|  | *Decline of cognitive function* | | | | | | | | |
| --- | --- | --- | --- | --- | --- | --- | --- | --- | --- |
|  | | List learning | List recall | List recognition | Story memory | Story recall | Figure recall | Immediate Memory | Delayed Memory |
| *Reduction of RNFL thickness in right eye* | | | | | | | | | |
| Average RNFL | | -0.34 (0.104) | -0.34 (0.121) | -0.08 (0.716) | 0.01 (0.970) | -0.18 (0.403) | 0.22 (0.312) | -0.19 (0.350) | -0.24 (0.227) |
| Superior RNFL | | 0.03 (0.889) | 0.10 (0.617) | 0.11 (0.594) | 0.14 (0.519) | -0.05 (0.821) | 0.04 (0.872) | 0.08 (0.706) | 0.17 (0.393) |
| Inferior RNFL | | -0.13 (0.581) | -0.41 (0.089) | 0.08 (0.742) | -0.35 (0.132) | -0.25 (0.296) | 0.16 (0.528) | -0.16 (0.494) | -0.42 (0.053) |
| Nasal RNFL | | **-0.44 (0.026)** | -0.23 (0.290) | **-0.41 (0.034)** | 0.27 (0.177) | 0.21 (0.292) | -0.04 (0.871) | -0.22 (0.279) | -0.17 (0.717) |
| Temporal RNFL | | -0.18 (0.108) | -0.19 (0.400) | 0.05 (0.803) | -0.06 (0.780) | -0.32 (0.124) | 0.35 (0.109) | -0.13 (0.551) | -0.21 (0.281) |
| *Decrease of hippocampus volume in right hemisphere* | | | | | | | | | |
| Hippocampus | | **-0.52 (0.010)** | -0.23 (0.291) | 0.22 (0.299) | -0.18 (0.390) | 0.34 (0.101) | -0.12 (0.594) | **-0.53 (0.008)** | 0.23 (0.246) |

Abbreviations: RNFL, retinal nerve fiber layer. The data were represented as standardized regression coefficient (*P* values), which were generated with multivariable linear regression adjusting for age, gender and education years. Significant results are indicated in bold.
